# Clinical Reports

**Published:** 1882-01

**Authors:** 


ARTICLE III.

### CLINICAL REPORTS.

### A NEW OPERATION FOR EXSECTION OF THE INFERIOR MAXILLARY NERVE IN THE SPHENO-MAXILLARY FOSSA.

At the Hospital of Oral Surgery, Philadelphia, an operation for this exsection was practiced by Prof. Garretson at his clinic, Nov. 22, which, so far as are concerned ease and certainty of the performance, places the matter in an entirely new position and converts complexity into simplicity.

After making the required trapway, by dissecting the masseter muscle from its attachment to the ramus of the lower jaw, a cylindrical bur, half an inch in length, was inserted into the mandril of a powerful surgical engine, and while in revolution to the extent of several thousand times in a minute, the nerve was quickly laid bare to its place of entrance into the bone at the posterior dental foramen. Next, the opening being enlarged until the pterygoid muscle was fairly exposed to view, the nerve was cut at the site of its inferior exposure, and being lifted from its bed and held on the stretch the handle of a scalpel was made to isolate it up to the point of emergence at the base of the skull. It was there excised, a pair of delicate Iris scissors being used.

The ease with which this most complex operation is performed after the manner described requires to be seen to be appreciated. The impression produced on the large number of students and medical gentlemen present was marked. It will surely divest the operation of the fear and hesitation always felt heretofore by one undertaking it.

